# Invariant Natural Killer T Cells Ameliorate Monosodium Urate Crystal-Induced Gouty Inflammation in Mice

**DOI:** 10.3389/fimmu.2017.01710

**Published:** 2017-12-12

**Authors:** Jie Wang, Qibin Yang, Quanbo Zhang, Congcong Yin, Li Zhou, Jingguo Zhou, Yangang Wang, Qing-Sheng Mi

**Affiliations:** ^1^Department of Endocrinology, Affiliated Hospital of Qingdao University, Qingdao, China; ^2^Immunology Research Program, Henry Ford Cancer Institute, Henry Ford Health System, Detroit, MI, United States; ^3^Center for Cutaneous Biology and Immunology Research, Department of Dermatology, Henry Ford Health System, Detroit, MI, United States; ^4^Department of Rheumatology, Affiliated Hospital of North Sichuan Medical College, Nanchong, China; ^5^Department of Gerontology, Affiliated Hospital of North Sichuan Medical College, Nanchong, China; ^6^Department of Internal Medicine, Henry Ford Health System, Detroit, MI, United States

**Keywords:** invariant natural killer T cells, gout, macrophage, polarization, tumor necrosis factor-α

## Abstract

Gout is an inflammatory arthritis caused by deposition of intra-articular monosodium urate (MSU) crystal. Previous studies have focused on resident macrophage, infiltrating monocyte, and neutrophil responses to MSU crystal; yet the mechanisms of cellular changes and the potential involvement of other regulatory immune cells remain largely unknown. Invariant natural killer T (*i*NKT) cells, an innate type of T cell, are involved in the development of various inflammatory diseases. Here, we investigate the role of *i*NKT cells in MSU crystal-induced gouty inflammation. MSU crystal-induced inflammatory profiles in an air-pouch model were examined in *i*NKT-deficient CD1d knockout (KO) and wild-type (WT) control mice. To explore potential mechanisms of *i*NKT cell regulation of gouty inflammation, we cocultured CD4^+^ or CD4^−^ *i*NKT cells with bone marrow-derived macrophages (BMDMs). We found that *i*NKT cells quickly migrated to the site of inflammation upon MSU crystal stimulation in WT mice. The total number of infiltrating cells in CD1d KO mice, especially neutrophils, was dramatically increased at 6 and 12 h (*P* < 0.01) post-MSU crystal challenge, compared with WT controls. BMDMs cocultured with CD4^+^ *i*NKT cells produced less tumor necrosis factor-α and expressed higher levels of M2 macrophage markers, including Clec7a, Pdcd1Ig2, and interleukin-4 (*P* < 0.01), compared with BMDMs cocultured with CD4^−^ *i*NKT cells or conventional CD4^+^ T cells. CD4^+^ *i*NKT cells are one of the key regulators of MSU crystal-induced gouty inflammation through the control of macrophage polarization. *i*NKT cells may serve as a new therapeutic target for gout.

## Introduction

Gout is a paradigm of acute, self-limited inflammation caused by the deposition of intra-articular monosodium urate (MSU) crystal (1). During the progression of the inflammatory response, MSU crystal provides both of extracellular and intracellular danger signals (2), whose recognition by toll-like receptor 2 (TLR2) and TLR4, as well as CD14, expressed on the surface of monocytes/macrophages would initiate MSU crystal uptake (3–5). Monocytes stimulated by MSU crystal can polarize into hyper-inflammatory M1 macrophages, which initiate inflammation *via* fully functional phagocytizing MSU crystal and delivering to cytoplasmic NACHT-LRR-PYD-containing protein-3 (NALP3) inflammasome, thereby producing tumor necrosis factor-α (TNF-α) and interleukin-1β (IL-1β), both are known as highly inflammatory cytokines, and promoting secondary neutrophil physiologic flow into the site of inflammation (6, 7). In contrast to M1 macrophages, anti-inflammatory M2 macrophages can dampen acute MSU crystal-induced inflammation and suppress caspase-1 activation and IL-1β production (8). The polarization from M1 into M2 macrophages in the development of gout may contribute to self-recovery. However, it is still unclear how this process is being regulated.

Invariant natural killer T (*i*NKT) cells are innate T cells that develop in the thymus and are selected by the MHC I homolog molecule CD1d on double-positive (DP) thymocytes (9). Based on their cytokine production profiles, *i*NKT cells are further subdivided into NKT1, NKT2, and NKT17, while the majority of CD4^+^ *i*NKT cells are NKT2 in mice, and they are activated by glycolipid antigens presented by CD1d on antigen-presenting cells (APCs) such as macrophages and dendritic cells. Upon activation, the subsets of *i*NKT cells differentially influence the immune response, producing either Th1 cytokines, such as interferon-γ (IFN-γ) and IL-2, or Th2 cytokines, such as IL-4 and IL-10 (10). Recently, a number of studies have reported that *i*NKT cells play a major role in mediating joint inflammation, including that of rheumatoid arthritis (11, 12). Nonetheless, the involvement of *i*NKT cells in gouty inflammation has yet to be clarified. In this study, we produced MSU crystal-induced gouty inflammation in a synovium-like subcutaneous air-pouch model using *i*NKT cell-deficient mice to identify the protective role of *i*NKT cells in MSU crystal-induced gouty inflammation. Our bone marrow-derived macrophage (BMDM)-*i*NKT coculture experiment indicates the regulatory role of *i*NKT cells in macrophage polarization, which could contribute to the protective function of *i*NKT cells in gouty inflammation.

## Materials and Methods

### Mice

CD1d knockout (KO) mice on a C57BL/6 background and wild-type (WT) C57BL/6 mice were purchased from the Jackson Laboratory and housed in a specific pathogen-free barrier unit. Experiments were conducted at 8–12 weeks of age and gender matched. Animal handling and the experimental procedures were approved by Institutional Animal Care and Use Committee of Henry Ford Health System.

### Subcutaneous Air-Pouch Model

**Figure 1:**
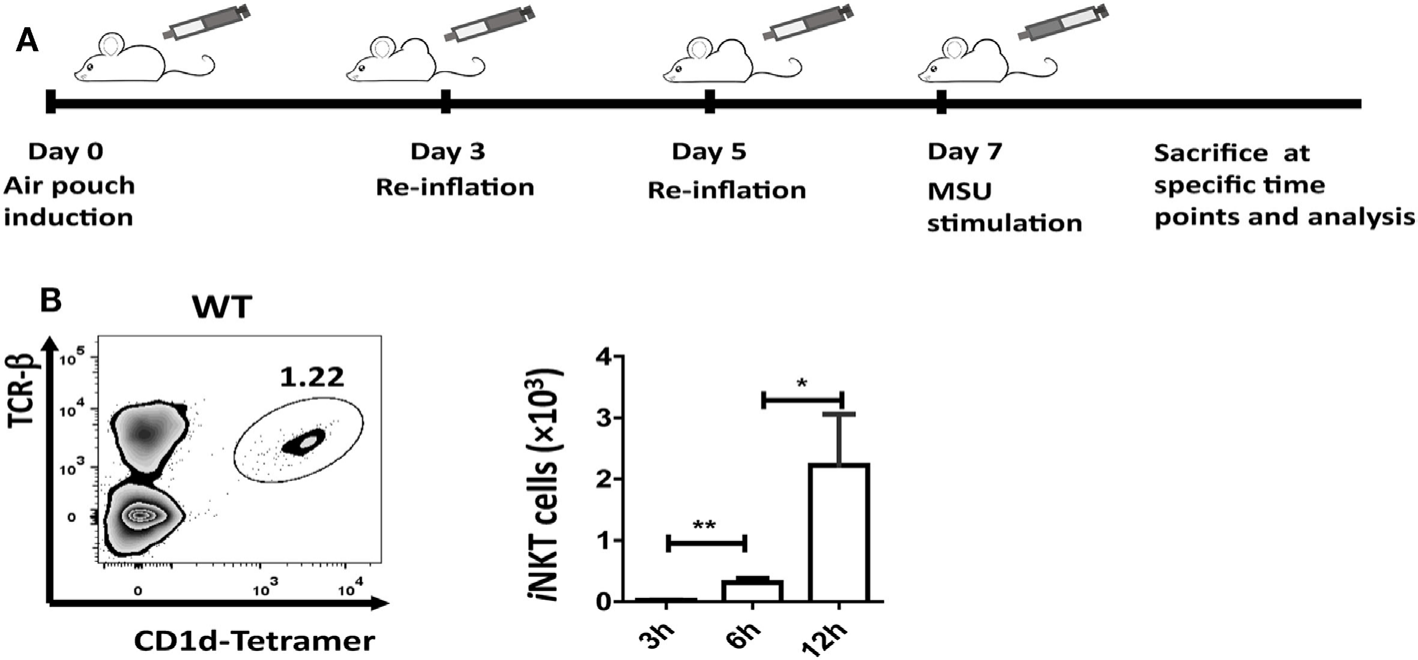
Invariant natural killer T (*i*NKT) cells are recruited to the site of inflammation induced by monosodium urate (MSU) crystal. **(A)** Outline of the synovium-like mouse subcutaneous air-pouch model. Subcutaneous air pouches were generated by injection of 5 mL air into the subcutaneous tissue of the back, followed by injection of another 3 mL of air at day 3 and day 5. At day 7, MSU crystal (3 mg in 1 mL) was injected into air-pouch cavities. **(B)** Flow cytometry analysis identified *i*NKT cells (TCRβ^hi^ and CD1d-tetramer^hi^) in wild-type (WT) air-pouch cavities at 12 h post-MSU crystal challenge (right panel); the bar graph shows the absolute number of *i*NKT cells infiltrating the air-pouch cavities at different time points (left panel). Results are representative of three independent experiments. Values are the mean and SEM (*n* = 3–4 per group). Significance for all data was determined by unpaired Student’s *t*-test: ns, not significant; *P ≤ 0.05; **P ≤ 0.01.

Injection of 5 mL of air into the subcutaneous tissue on the back of mice was followed by injection of an additional 3 mL of air on day 3 and day 5. On day 7, MSU crystal (3 mg in 1 mL) was injected into the air-pouch cavities, and cells were harvested with 2 mL PBS at 3, 6, or 12 h for flow cytometry and enzyme-linked immunosorbent assays (ELISA) analyses (Figure 1A).

### Purification of *i*NKT and CD4^+^ T Cells

Total spleen cells from WT mice were first stained with anti-mouse CD8 biotin and anti-mouse B200 biotin Abs, then CD8^+^ and B220^+^ cells were depleted with anti-biotin beads using auto MACS (Miltenyi Biotec). Negatively selected cells were then stained with anti-mouse TCR-β, anti-mouse CD4 Ab, and PBS57-CD1d tetramers. CD4^+^ *i*NKT cells, CD4^−^ *i*NKT cells, and CD4 T cells (>97% purity) were sorted by BD FACS Aria II.

### *i*NKT–BMDM Coculture Assay

BM cells from WT or CD1d KO mice were incubated in culture media (RPMI with 10% FBS and 30 ng/mL M-CSF) for 7 days to induce BMDMs (M0). BMDMs were pre-loaded with α-galactosylceramide (α-GalCer, 1 µg/mL) for 6 h before they were cocultured with sorted CD4^+^ *i*NKT cells, CD4^−^ *i*NKT cells, or CD4^+^ T cells, respectively, at a 2:1 ratio for 36 h, followed by stimulation with MSU crystal (50 µg/mL) for 4 h with or without *i*NKT cells. After MSU crystal stimulation, BMDMs were collected for flow cytometry analysis or qRT-PCR.

### Flow Cytometry

Single-cell suspensions were washed twice with staining buffer and incubated with Fc block (clone 2.4G2). The following conjugated mAbs were used for flow cytometry analysis: TCR-beta (H57-597), PBS57-CD1d tetramers, F4/80 (BM8), Ly6G (1A8), and TNF-α (MP6-XT22). Dead cells were first gated out by propidium iodide (PI) staining. All mAbs were purchased from BD biosciences or eBioscience. Data were analyzed using FlowJo software.

### MSU Phagocytosis of Macrophages

The peritoneal cells were harvested at 6 h after MSU crystal (3 mg in 0.5 mL) treatment through peritoneal cavity. The phagocytosis of MSU crystals was determined by analyzing the side scatter (SSC) change in the flow cytometry.

### RNA Extraction and Quantitative RT-PCR

Total RNA was extracted from BMDMs using a Mammalian Total RNA Miniprep Kit (Sigma). Q-PCR data collected on the QuantStudio 7 were normalized to the β-actin gene in the corresponding sample. Primer sequences are listed in Table S1 in Supplementary Material.

### Enzyme-Linked Immunosorbent Assays

Cytokines in the supernatant of air-pouch cavities were measured using the ELISA Ready-Set-Go kit (eBioscience).

### Statistical Analysis

Statistical analysis was performed with Prism 7.0 (GraphPad Software). The two-tailed Student’s *t*-test was used. Differences were considered statistically significant when *P* < 0.05.

## Results

### *i*NKT Cells Are Quickly Recruited into the Inflammatory Site Induced by MSU Crystal

The air-pouch inflammation model was used for the investigation of synovial inflammation-mediated by MSU. To determine whether *i*NKT cell recruitment is a general feature in MSU crystal-induced gouty inflammation, cellular components in the washing fluid of the air-pouch cavity were analyzed by flow cytometry at different time points post-MSU crystal challenge. The *i*NKT cell population was identified by CD1d-tetramer and anti-TCRβ staining (Figure 1B). To our surprise, *i*NKT cells were recruited into the air-pouch cavities starting at 3 h and reached the peak at 12 h post-MSU crystal challenge (Figure 1B). Thus, it is reasonable to speculate that the *i*NKT cells recruited to sites of inflammation may have a role in the regulation of gout development.

### *i*NKT-Deficient Mice Developed More Severe MSU Crystal-Induced Inflammation

**Figure 2:**
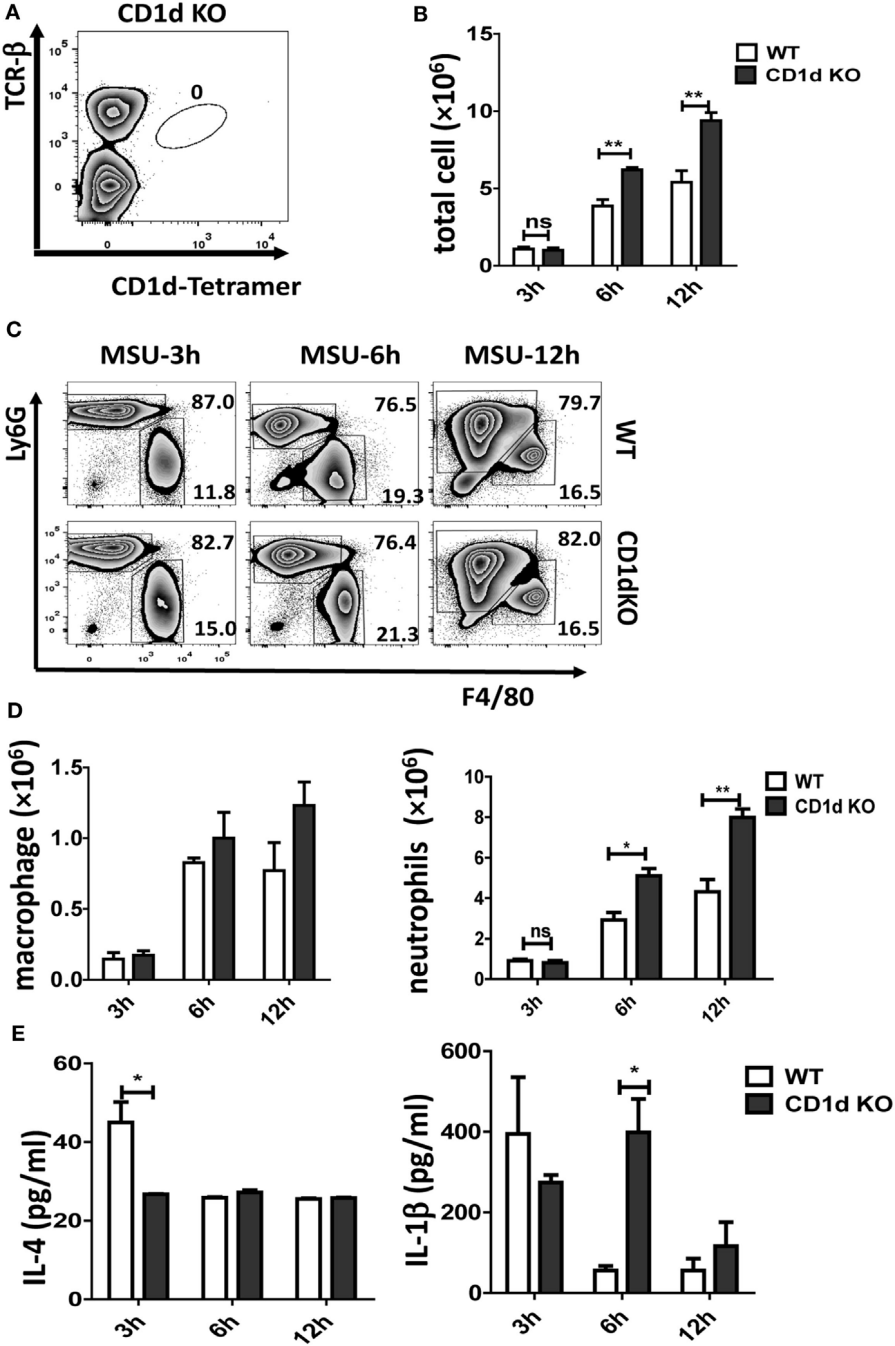
Deletion of invariant natural killer T (*i*NKT) cells enhances the monosodium urate (MSU) crystal-induced inflammatory response. **(A)** Flow cytometry analyses of *i*NKT cells from CD1d knockout (KO) air-pouch cavity at 12 h post-MSU crystal challenge; **(B)** a bar graph showing the total cell numbers from air-pouch cavities in wild-type (WT) and CD1d KO mice at different time points after MSU crystal treatment; **(C)** flow cytometry analyses of macrophages (Ly6G^−^F4/80^hi^) and neutrophils (Ly6G^hi^F4/80^−^) from air-pouch cavities based on the expression of Ly6G and F4/80 at different time points post-MSU crystal treatments. **(D)** The absolute numbers of infiltrating macrophages (left) and neutrophils (right) in the air-pouch cavities from WT mice (white) and CD1d KO mice (gray) over 12 h. **(E)** Supernatant from air-pouch cavities was analyzed for interleukin-4 (IL-4) and interleukin-1β (IL-1β) by enzyme-linked immunosorbent assays. Results are representative of three independent experiments. Values are the mean and SEM (*n* = 3–4 per group). Significance for all data was determined by unpaired Student’s *t*-test: ns, not significant; *P ≤ 0.05; **P ≤ 0.01.

CD1d is required for *i*NKT cell selection in the thymus and CD1d KO mice fail to develop *i*NKT cells. As shown in Figure 2A, there is no *i*NKT cell migration into air-pouch cavities of CD1d KO mice upon MSU crystal stimulation. To understand the function of *i*NKT cells in MSU crystal-induced gouty inflammation, we examined inflammatory profiles in WT mice and *i*NKT-null CD1d KO mice using an air-pouch gouty model. Upon MSU crystal stimulation, the total inflammatory cell number in the washing fluid from air-pouch cavities was significantly increased in CD1d KO mice compared with WT mice at 6 h (*P* < 0.01) and 12 h (*P* < 0.01) post-MSU crystal challenge (Figure 2B). We next evaluated neutrophils and macrophages in the washing fluid of air-pouch cavities by flow cytometry (Figure 2C). Even though the frequencies of neutrophils were not significantly increased, the absolute number of neutrophils increased dramatically (*P* < 0.05 at 6 h, *P* < 0.01 at 12 h) in CD1d KO mice. Nevertheless, no significant changes in frequency or number of macrophages were observed (Figure 2D). Therefore, the mice without *i*NKT cells developed more severe MSU crystal-induced inflammation.

Next, the levels of IL-4 and IL-1β in the supernatants from air-pouch cavities were analyzed by ELISA. As expected, both cytokines were elevated at 3 h following MSU crystal treatment in WT mice, then quickly downregulated at 6 and 12 h (Figure 2E). Interestingly, the level of IL-1β was dramatically increased in CD1dKO mice compared with WT mice at 6 h, whereas IL-4 was significantly reduced in CD1dKO mice at 3 h post-MSU crystal challenge. Thus, *i*NKT cells may regulate cytokine production, reducing IL-1β but increasing IL-4 production by macrophages. Taken together, these results suggest that *i*NKT cell deficiency significantly enhances the inflammatory response to MSU crystal *in vivo*.

### Lack of CD1d in Macrophages Does Not Affect Their MSU Crystal Phagocytosis

**Figure 3:**
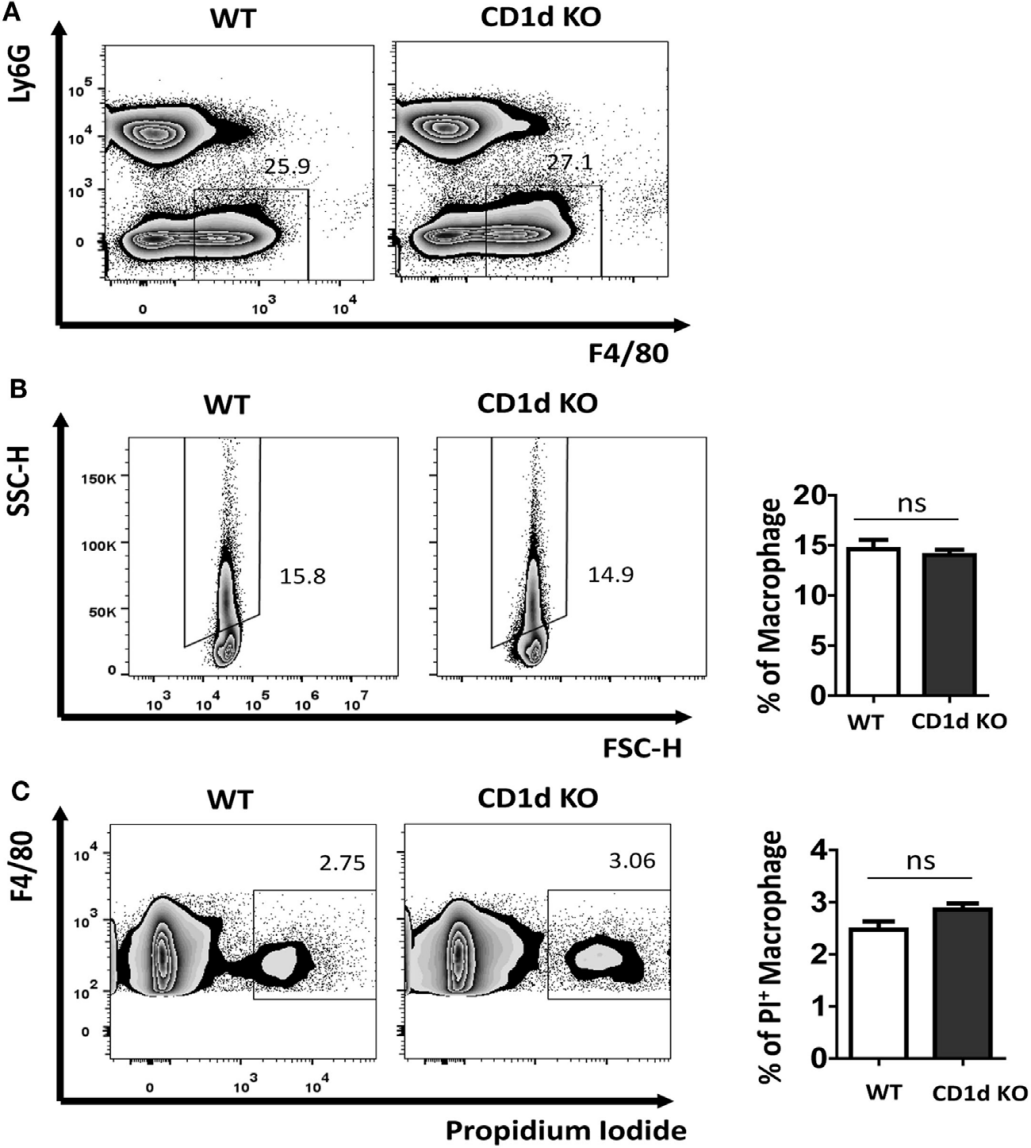
Comparable monosodium urate (MSU) crystal phagocytosis in macrophage between wild-type (WT) and CD1d knockout (KO) mice. Flow cytometry first gated peritoneal macrophages (Ly6G^−^F4/80^hi^) **(A)** and then analyzed their side scatter (SSC) increase **(B)** and PI^+^ dead cells **(C)** at 6 h post-MSU crystal treatment. The bar graph showed the frequencies of SSC increased macrophages **(B)** and PI^+^ macrophages **(C)**. Results are representative of three to four mice per group. Values are the mean and SEM. ns, not significant.

The uptake of MSU crystal by macrophages is a feature for recognition by NALP3 inflammasome, thereby triggering caspase-1 activation and IL-1β processing (7, 13). Recently, Liu-Bryan et al. found that CD14 mediated ingestion of MSU crystal was able to induce an inflammatory response, leading to IL-1β release, which, however, is independent of modulation of MSU crystal uptake (5, 14). To explore if the increased production of IL-1β in CD1d KO air-pouch cavities was due to the modulation of capacity of MSU crystal uptake in the deletion of *i*NKT cells, we next preformed macrophage MSU crystal uptake assay *in vivo*. WT and CD1d KO mice were challenged with MSU crystal peritoneally for 6 h. Subsequently, the macrophages (Ly6G^−^F4/80^hi^) were further analyzed for their SSC change, whose increasing would reflect the uptake of MSU crystal. As shown in Figures 3A,B, MSU crystal phagocytosis were comparable between WT and CD1d KO mice, which indicated that MSU crystal-induced severe inflammation, including high production of IL-1β, observed in CD1d KO mice is not due to increased MSU crystal phagocytosis. Thus, CD1d deficiency does not affect phagocytosis. Next, to rule out the possibility that lack of iNKT cells may affect macrophage death during MSU crystals induced gouty inflammation, we did a cell viability assay. As shown in Figure 3C, the frequency of PI^+^ dead macrophages was comparable between WT and CD1d KO mice. Thus, lack of *i*NKT cells unlikely affect MSU crystal induced macrophage death.

### CD1d Deficiency Does Not Affect Macrophage Function

Since CD1d is also expressed in APCs, including macrophages, to exclude the possibility that the exacerbation of MSU crystal-induced gouty inflammation in CD1d KO mice was due to the lack of CD1d in the macrophages, we examined the TNF-α production by CD1dKO BMDMs using flow cytometry. As shown in Figure S1 in Supplementary Material, the frequencies of TNF-α secreting BMDMs from WT and CD1d KO mice were comparable post-MSU crystal stimulation. Thus, CD1d deficiency in macrophages may not affect their cytokine production in response to MSU crystal. Therefore, deficiency of *i*NKT cells in CD1d KO mice likely contributes to enhanced gout development.

### Activation of CD4^+^*i*NKT Cells Inhibits MSU Crystal-Induced Gouty Inflammation

**Figure 4:**
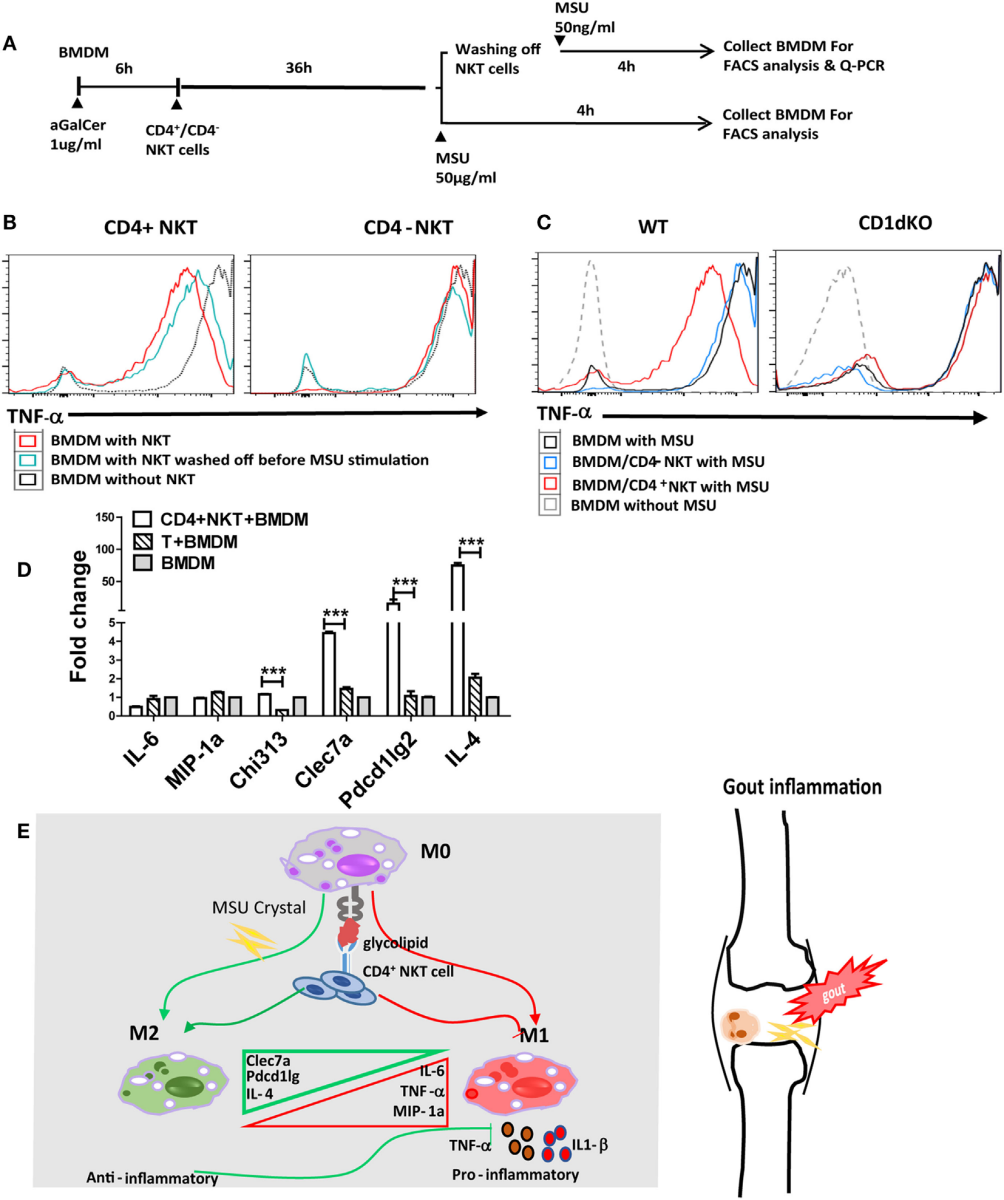
Activated CD4^+^ invariant natural killer T (*i*NKT) cells suppress monosodium urate (MSU) crystal-induced inflammation by promoting M2 polarization. **(A)** Outline of the bone marrow-derived macrophage (BMDM) and *i*NKT cell coculture system. BMDMs were cultured in media (RPMI with 10% FBS) with or without the presence of α-galactosylceramide (α-GalCer) (100 ng/mL, 6 h), cocultured with CD4^+^ *i*NKT or CD4^−^ *i*NKT cells for 36 h, then treated with MSU crystal for 4 h with or without *i*NKT cells. **(B)** BMDMs, cultured with CD4^+^ or CD4^−^ *i*NKT cells and treated with MSU crystal, were analyzed by flow cytometry for the presence of tumor necrosis factor-α (TNF-α)-producing BMDM. **(C)** BMDM from wild-type (WT) or CD1d knockout (KO) mice, cultured with CD4^+^ or CD4^−^ *i*NKT cells and treated with MSU crystal, were analyzed by flow cytometry for the presence of TNF-α positive BMDM. **(D)** Q-PCR analysis of M1 (TNF-α, IL-6, and MIP-1a) and M2 [Clec7a, pdcd1Ig2, and interleukin-4 (IL-4)] genes in BMDMs from WT mice cocultured with CD4^+^ *i*NKT cells, compared with CD4^+^ T cells or BMDM alone. **(E)** The potential working model of *i*NKT cells in gouty inflammation.

Given that *i*NKT cells present quite early in sites of inflammation and may interact with CD1d^+^ APCs, macrophages in particular, we raised the hypothesis that the cross talk between *i*NKT cells and macrophages may interrupt MSU crystal-induced inflammation. There are at least two subsets of *i*NKT cells, such as CD4^+^ and CD4^−^ *i*NKT cells, which seem to possess different functions in regulating disease development (15). To investigate the contribution of *i*NKT cells to the cellular events at the site of gouty inflammation, we performed the *in vitro i*NKT cell–BMDM coculture experiment. BMDMs from CD1d KO or WT mice were cocultured with either sorted CD4^+^ or CD4^−^ splenic *i*NKT cells, and treated with α-Galcer, an agent to activate *i*NKT cells (16). BMDMs were collected after MSU crystal stimulation, with or without the washing off of *i*NKT cells before MSU crystal stimulation (Figure 4A). As shown in Figure 4B, BMDMs from WT mice cocultured with CD4^+^ *i*NKT cells produced less TNF-α upon MSU crystal stimulation than macrophages not cocultured with CD4^+^ *i*NKT cells. Coculture with CD4^−^ *i*NKT cells did not dramatically change TNF-α production compared with the BMDMs alone. This result suggests that it was CD4^+^ *i*NKT cells rather than CD4^−^ *i*NKT cells that played a key role in controlling gouty inflammation. As expected, coculture of *i*NKT cells with the BMDMs from CD1d KO mice, which are unable to stimulate *i*NKT cells due to their lack of the CD1d molecule, showed that neither CD4^−^ nor CD4^+^ *i*NKT cells inhibited TNF-α production (Figure 4C), which suggests that the activation of *i*NKT cells is required to inhibit TNF-α production in BMDMs. Thus, CD4^+^ *i*NKT cells regulate gouty inflammation in a CD1d-dependent manner.

### Activation of CD4^+^*i*NKT Cells Enhance M2 Macrophage Polarization in MSU Crystal- Induced Gouty Inflammation

Previous studies have suggested that activated *i*NKT cells modulate macrophage differentiation from M1 to M2 macrophages (17–19). To test if *i*NKT cells can regulate gouty inflammation through macrophage polarization, we assessed macrophage polarization-related cell markers using qRT-PCR analysis. As shown in Figure 4D, coculture with CD4^+^ *i*NKT cells significantly upregulated M2-related gene expressions on BMDMs, including Clec7a (*P* < 0.01), Pdcd1Ig2 (*P* < 0.01), and IL-4 (*P* < 0.01), compared with that of BMDMs cultured alone or cocultured with CD4^+^ conventional T cells; whereas expression levels of M1 markers (IL-6 and MIP-1a) were unaffected. Collectively, activation of CD4^+^ *i*NKT cells promotes M2 polarization, which may contribute to *i*NKT cell-mediated protection from MSU crystal-induced gouty arthritis.

## Discussion

To the best of our knowledge, this is the first study to show that *i*NKT cells are actively involved in the development of gouty inflammation. We found that post-MSU crystal stimulation, *i*NKT cells quickly migrate to the site of inflammation, and that *i*NKT cell deficiency results in more severe MSU crystal-induced gouty inflammation in mice. Thus, early *i*NKT cell migration to the site of inflammation is required to control gouty inflammation, which could be a key mechanism contributing to gout self-recovery. A previous study strongly suggested that CD4^+^ *i*NKT cells mainly produce Th2 cytokines, such as IL-4 and IL-10; and control the development of autoimmune and inflammatory diseases (20). In our study, the CD4^+^, but not the CD4^−^, subset of *i*NKT cells can reduce macrophage TNF-α production in response to MSU crystal, which further supports previous notions. The cross talk between *i*NKT cells and macrophages has recently been identified in different diseases (17, 21–25). *i*NKT cells have the potential to promote macrophage polarization to the M2 subtype in adipose tissue and are involved in the development of metabolic syndrome, such as obesity, insulin resistance, and diabetes (17–19, 26). Recently, more evidence supports the notion that hyperuricemia and gout are tightly linked to metabolic syndrome (27). Consistent with previous findings in metabolic syndrome, we found that CD4^+^ *i*NKT cells can enhance M2 polarization, which may contribute to protection against MSU crystal -induced inflammation.

Accumulated clinical studies suggest that *i*NKT cell number and subset derangements are related to the development of autoimmune and inflammatory diseases (11, 12). Thus, it is worthwhile to further investigate *i*NKT cell number and function in gout. Based on current findings, we suggest the potential working model for the involvement of *i*NKT cells in gouty inflammation described in Figure 4E. Overall, *i*NKT cells quickly migrate to inflammatory site upon MSU crystal stimulation, and activation of CD4^+^ *i*NKT cells control MSU crystal-induced gouty inflammation by promoting M2 macrophage polarization. Our data strongly suggest that CD4^+^ *i*NKT cells are one of the key regulators in the control of MSU crystal-induced gouty inflammation, and that *i*NKT cells may serve as a new therapeutic target for gout.

## Ethics Statement

This study was carried out in accordance with the requirements of Institutional Animal Care and Use Committee.

## Author Contributions

JW and QY performed most of the experiments; QZ performed some key experiments; CY maintained and genotyped mutant mice; Q-SM, LZ, JW, QY, and QZ analyzed the data; JW and Q-SM drafted the manuscript; Q-SM, YW, and JZ supervised the overall study and finalized the manuscript.

## Conflict of Interest Statement

The authors declare that the research was conducted in the absence of any commercial or financial relationships that could be construed as a potential conflict of interest.
