# Supplementary material for: Invariant Natural Killer T Cells Ameliorate Monosodium Urate Crystal-Induced Gouty Inflammation in Mice
Source: Front Immunol. 2017 Dec 12;8:1710. doi: 10.3389/fimmu.2017.01710 (PMC5733058; doi:10.3389/fimmu.2017.01710)
Supplement: Supplementary file 1 [file table_1.PDF]

Supplementary Material

Invariant Natural Killer T Cells Ameliorate Monosodium Urate Crystal- Induced Gouty Inflammation in Mice

Jie Wang, Qibin Yang, Quanbo Zhang, Congcong Yin, Li Zhou, Jingguo Zhou, Yangang Wang, and Qing-Sheng Mi

Supplementary Table 1: RT-PCR Primers used in this study

Inflammatory gene analysis		
Target genes	Sequence F	Sequence R
Interleukin 4(IL-4)	CATGGGAAAACTCCATGCTT	TGGACTCATTCATGGTGCAG
TNF-a (TNF-a)	TCAGCCGATTTGCTATCTCATA	AGTACTTGGGCAGATTGACCTC
Interleukin6(IL-6)	AGACAAAGCCAGAGTCCTTCAG	TGCCGAGTAGATCTCAAAGTGA
MIP-1a(Mip1a)	TTCTCTGTACCATGACACTCTGC	CGTGGAATCTTCCGGCTGTAG
Arginase 1(Arg1)	CTCCAAGCCAAAGTCCTTAGAG	AGGAGCTGTCATTAGGGACATC
Programmed cell death 1 ligand 2 (Pdc1lg2)	ACGTGGCCACTTCATGTTTT	TCTTGAGGGTTTCCCATCAG

Supplementary Figure 1.

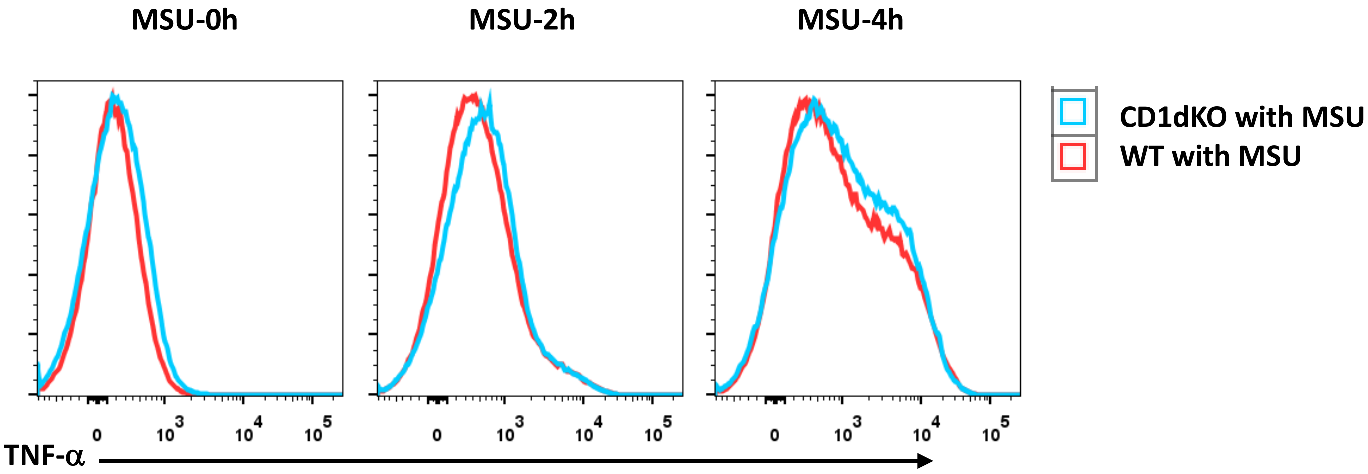

Supplementary Figure 1: TNF-α producing BMDMs from WT (red) and CD1d KO mice (blue) upon MSU crystals stimulation at 2 hours and 4 hours post MSU crystal stimulation.
